# Avoidance Behaviour of Six Collembolan Species Shows Species-Specific Sensitivity—Impact of Ag NM300K

**DOI:** 10.3390/nano12193276

**Published:** 2022-09-21

**Authors:** Marija Kovačević, Mónica J. B. Amorim, Branimir K. Hackenberger, Janeck J. Scott-Fordsmand

**Affiliations:** 1 Department of Biology, University of Osijek, Cara Hadrijana 8A, HR 31000 Osijek, Croatia; 2 Department of Biology & CESAM, University of Aveiro, 3810-193 Aveiro, Portugal; 3 Department of Ecoscience, Aarhus University, C.F. Møllers Alle 3, DK-8000 Aarhus, Denmark

**Keywords:** biodiversity, sustainability, green deal, collembola, species sensitivity distribution, life-traits

## Abstract

Although standard testing guidelines use a species as a representative surrogate, species-specific sensitivity is well-known. The aim of this study was to investigate the species-specific difference in avoidance behaviour among Collembola species exposed to silver (Ag) nanomaterials (NM) (Ag NM300K). The avoidance test was performed with *Folsomia candida*, an international standard species in laboratory tests, and five widely distributed species with different life history traits, commonly used in small multispecies systems (*Folsomia fimetaria*, *Proisotoma minuta*, *Mesaphorura macrochaeta*, *Protaphorura fimata* and *Ceratophysella denticulata*). There was higher avoidance in euedaphic species, such as *F. candida* and *F. fimetaria*, compared to the epiedaphic species *C. denticulata*, which showed the least avoidance behaviour. An explanation may be that euedaphic species (living in deeper soil layers) are more directly exposed within the soil pores and have developed a pronounced avoidance behaviour. In contrast, species living on the surface are likely less directly exposed and hence only avoid at higher total concentrations. Additionally, difference in cuticula between the groups, providing different degrees of protection against exposure, can explain the different behaviours. The present results highlight the importance of biodiversity for the ecosystem and raise awareness on species sensitivity.

## 1. Introduction

Collembola is the most abundant class of soil arthropods, occupying various niches in soil ecosystems [1]. Due to their high morphological and ecological diversity, collembolans can play different roles in soil ecosystems [2]. In addition to their influence on various processes in soil ecosystems, collembolans, especially large surface-dwelling species, are an important food source for a large number of soil invertebrates [1]. Evolution has led to differentiation of roles among species, including different physiologies, life strategies and, perhaps, sensitivities to environmental clues, e.g., in avoiding contaminants in the environment [3]. An inability to avoid hazardous contaminants can obviously lead to higher risk for population extension [4,5]. Therefore, in addition to survival, reproduction and various molecular biomarkers, it is important to determine the effect of individual substances on avoidance behaviour.

Due to their widespread use, nanomaterials (NMs) are among the contaminants that are increasing in the ecosystem. Soils and terrestrial ecosystems are some of the main sinks for NMs [6], and NMs may affect non-target organisms in soil. Silver nanomaterials (AgNM) are widely used and have well known inherent toxicity (e.g., anti-microbial). AgNM are particularly important for medicine and cosmetics, but also for industries such as electronics, packaging or textiles and plastics manufacturing [7]. However, when released from consumer products during usage and at the end of their life [8,9,10], AgNM pose a direct threat to soil ecosystems and their inhabitants. Other studies have shown that AgNM can be detected and avoided by soil invertebrates, e.g., earthworms [11]. However, it is not known whether collembolans can avoid NM300K.

Various ecotoxicological tests are used to assess the toxicity of AgNM in soil organisms [12,13,14,15]. The ecotoxicological effects of AgNM on soil arthropods have been less studied than other soil organisms. The few studies that exist show negative effects, e.g., on reproduction and induction on various molecular biomarkers [16,17,18,19]. The species used in the standardised guidelines are very important and good representatives, although they do not cover the wide diversity among collembolans. Therefore, it is important to assess the impact of stressors on more species, and very few studies cover this aspect. One of the few examples of different species being tested is the Soil Multispecies Test System (SMS), which uses multiple species in a mesocosm [18,20,21,22,23].

The avoidance test is an important ISO test [24] and is commonly used for the collembolan *Folsomia candida* in relation to chemical hazard assessment. Traditionally, individual species, such as *F. candida*, are used as a representative of the many other species of collembolans, but it is clear that there are important differences between species, likely also in avoidance behaviour. Avoidance behaviour may vary between species due to differences in morphological, physiological and behavioural characteristics, in addition to differences in exposures. For example, some collembolan species forage in a distance up to 40 cm [25,26], while other species can forage up to several hundred metres [27]; moreover, some species live on the surface, while others live at various depths/fractions in the soil layers, i.e., epi-, hemi- and euedaphics, which depends on their morphology and physiology.

Considering this, the present study aimed to fill a knowledge gap and assess the species-specific avoidance behaviour of springtails towards Ag NM300K, a reference AgNM [28]. In addition to *F. candida*, a standard species in laboratory tests, five other species with different life history characteristics commonly used in SMS were used.

## 2. Materials and Methods

### 2.1. Test Organisms

**Figure 1:**
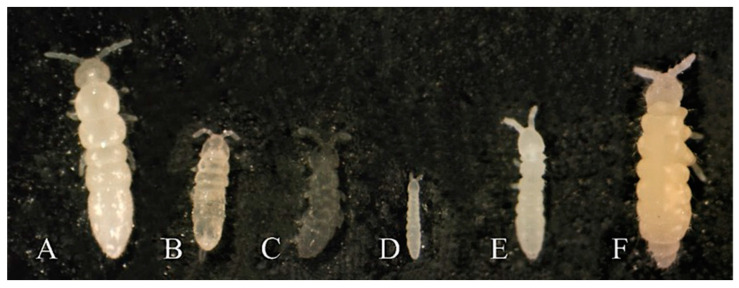
Collembolan species used in the avoidance test: *Folsomia candida* (**A**), *Folsomia fimetaria* (**B**), *Proisotoma minuta* (**C**), *Mesaphorura macrochaeta* (**D**), *Protaphorura fimata* (**E**) and *Ceratophysella denticulata* (**F**).

Six collembolan species were used: *Folsomia candida*, *Folsomia fimetaria*, *Proisotoma minuta*, *Mesaphorura macrochaeta*, *Protaphorura fimata* and *Ceratophysella denticulata* (Figure 1, Table 1). The animals were maintained in culture on a moist substrate plaster of Paris and activated charcoal under controlled conditions (19 ± 1 °C, 16:8 (light:dark) photoperiod). The organisms were fed with dried baker’s yeast once a week.

Adult organisms from each species were transferred on fresh substrate for egg laying. After 10 days, eggs were carefully collected, moved to egg paper and placed in fresh substrate. Two days after the start of hatching, the egg paper was removed, ensuring the same age of the juveniles. Juveniles (10–12 days old) were collected by suction and released into a small container with moist substrate (20 per replicate) to check their physical condition under the stereomicroscope (injured animals were disposed).

### 2.2. Test Soil

The standard natural soil LUFA 2.2 (LUFA Speyer, Speyer, Germany) was used. The main characteristics of the soil are a pH of 5.5 (0.01 M CaCl_2_), a cation exchange capacity (CEC) of 10.1 meq/100 g, 1.77% organic matter, a maximum water holding capacity (WHC) of 45.8%, and a grain size distribution of 7.3% clay, 13.8% slit and 78.9% sand. Prior to beginning the experiment, the soil was dried at 80 °C.

### 2.3. Test Material and Spiking

The standard reference nanomaterial Ag NM300K from JRC (Joint Research Centre, Ispra, Italy) was used [28]. In a short, a nominal silver content of 10.16% *w*/*w* was dispersed in 4% *w*/*w* polyoxyethylene glycerol trioleate and polyoxyethylene sorbitan mono-laurat (Tween 20). Ag NM300K were spherical particles without a coating, with 99% of particles having a nominal size of about 15 nm.

Spiking was performed via aqueous dilutions of Ag NM300K, added individually to each replicate of pre-moistened soil (25% *w*/*w*). The Ag NM300K was homogeneously mixed into the soil, and the remaining water needed to reach 50% WHC was added. The soil equilibrated for 24 h before the start of the test. The tested concentrations were 0, 5, 12.5, 25, 50, 75, 100, 150, 200, 400, 600 and 800 mg Ag NM300 K/kg soil DW (dry weight), after an initial optimization pre-test step.

### 2.4. Materials Characterization

Silver concentrations were measured in the soil samples with flame atomic absorption spectroscopy (AAS, Perkin Elmer 4100, Ueberlingen, Germany). The soil was prepared following the procedure of Scott-Fordsmand et al. [29]. Detailed characterization of the particles can be found in [28].

### 2.5. Experimental Procedure

The avoidance test was conducted according to the standardised ISO guideline [24]. Round plastic boxes (Ø 8 cm × 5 cm) with a removable barrier were used to divide them into two equal parts. One test compartment was filled with 30 g of control soil and the other with 30 g of spiked soil. After adding the soil, the plastic barrier was carefully removed. At the contact line between the two compartments, 20 synchronized age organisms were introduced. An EC_x_ design was carried out, i.e., a high number of treatments (12) and one replicate. The test was conducted for 48 h under controlled conditions (20 ± 1 °C, 16:8 h light:dark). At the end of the exposure period, the barrier was reinserted to separate the soil. The vessel was flooded with water, and the animals floated on the surface and were counted on each side. Each species was tested separately.

### 2.6. Data Analysis

The percentage of avoidance behaviour (A) was calculated as the difference between the number of organisms in the control soil (C) and the spiked soil (T) divided by the total number of organisms per replicate (N) (% A = ((C − T)/N) × 100). The avoidance effect concentrations (EC_x_) were calculated using the four-parameter logistic model were performed using data package drc [30] in the statistical software R 3.4.0 (R Core Team, Vienna, Austria) [31,32]. The species sensitivity distributions (SSDs) and the 5% hazard concentration (HC_5_) calculations were conducted using package ssd tools and log normal distribution, and were based on the EC_10_.

## 3. Results

The background level of Ag in LUFA 2.2 soil (control) was below the detection limit (0.005 mg Ag/kg), and the spike confirmed. The pH did not change significantly between tested concentrations and was 5.6 ± 0.1 (AV ± SD).

**Figure 2:**
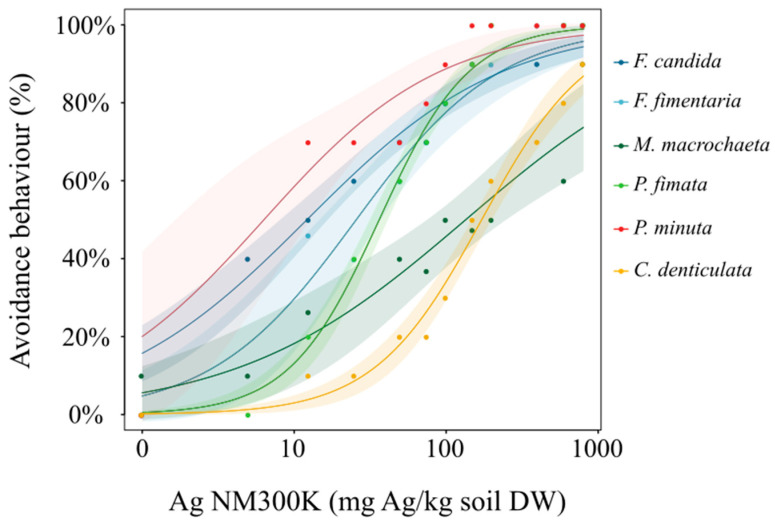
Avoidance behaviour for the different collembolan species when exposed to Ag NM300K (mg Ag NM300K/Kg soil DW) in LUFA 2.2 soil. Solid line represents the fitted model, the light section is the 95% confidence interval.

No mortality was observed during the avoidance test, and 100% of the animals were recovered. Controls showed a random distribution of animals on each side, excluding the possibility of aggregation tendency observed in some species or other artefacts. Exposure to Ag NM300K elicited a response in all tested species (Figure 2). The estimated avoidance effect concentrations (EC_x_) (Table 2) showed the differences in avoidance behaviour between tested species.

**Figure 3:**
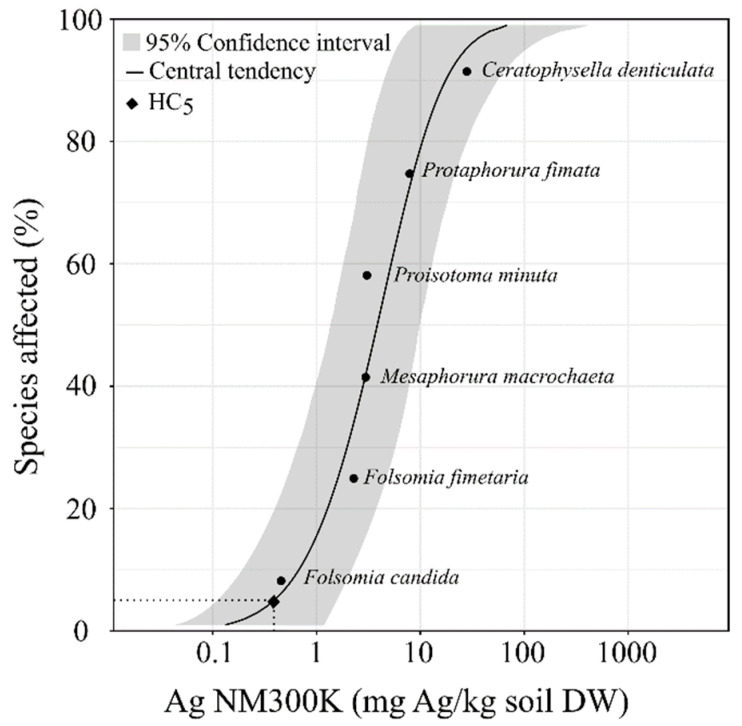
Species sensitivity distribution (SSD) based on chronic avoidance toxicity values (EC_10_) of six collembolan species exposed to Ag NM300K in LUFA 2.2 soil. Solid line represents the fitted log normal model, the grey section is the 95% confidence interval, and the dashed line represents hazardous concentrations for 5% of collembolan species (HC_5_).

Based on the above EC_10_ values, the calculated species sensitivity distribution (SSD) (Figure 3) and HC5 for the collembolan species was 0.39 (0.13–1.62) mg Ag/kg (r^2^ = 0.79).

## 4. Discussion

All tested collembolan species showed the ability to avoid Ag NM300K. The avoidance was in the same concentration range as observed for Ag NM300K in earthworms [11]. The shape of the SSD curve did not indicate an obvious species deviating in sensitivity. Many characteristics may be related to avoidance behaviour following exposure to contaminants, with habitat being an obvious determinant as it relates to morphology, physiology, feeding preference, exposure regimes, etc. One trait, the furca, has been considered a route of uptake for contaminants [33]. In the present study, we see that the furca trait matched with differences in avoidance behaviour, although this is not entirely clear. The highest avoidance behaviour (i.e., lowest EC_10_) was observed in *F. candida* and *F. fimetaria*, euedaphic species of the Isotomidae family that have a well-developed furca. These results are consistent with previous studies that identify *F. candida* as a metal-sensitive species [33]. This group mainly includes small species that reside in the soil with a larger surface ratio (size vs. surface), and they interact directly with the soil through feeding and soil pore water [34]. These species usually have complex post-antennal and antennal organs, which may play an important role in chemoreception [35]. If the furca is an important route of exposure, it makes sense that for species with reduced locomotor organs, i.e., furca, legs are less sensitive. The tested euedapic Onychiurinae family (*M. macrochaeta* and *P. fimata*), which are without furca, were characterised by lower avoidance behaviour (i.e., higher EC_10_) compared to the two other euedaphic species tested. *M. macrochaeta* was the smallest of all tested species, which could be the reason why it was more sensitive than *P. fimata*, the latter of which does not jump when disturbed, but rather curls up [36]. It is noticeable that *M. macrochaeta* has a flatter concentration–response curve compared to the other species (i.e., a larger difference between EC_10_ and EC_50_); the reason for this is unknown to us. Although the outer cuticle is less likely to be a major route of AgNM uptake, it is worthwhile remembering that eu-, hemi- and epiedaphic species may all differ in the cuticle structure, e.g., with euedaphic species having secondary granules and others not having them [37]. The hemiedaphic species *P. minuta*, which is associated with the upper litter layers and the soil surface [38] and with well-developed furca, could be sensitive because of the furca. Hence, as with the other species with furca, if they can detect a contaminant faster, they can better avoid direct exposure compared to species that do not have furca. Therefore, they would perhaps have less evolutionary pressure to develop internal defence mechanisms. The epiedaphic species *C. denticulata*, a large-bodied surface-dweller showed the least avoidance behaviour, i.e., the highest EC_10_. This is probably the result of high metal tolerance [39] and lower exposure to AgNM due to living on the soil surface [33]. Finally, the avoidance observed in the NM300K exposures could potentially be directly related to the concentration of NM300K or dissolved Ag, either through dissolution of NM300K to release “free Ag” ions, or the release of Ag(I) bound to the dispersant in the original NM300K [40,41].

## 5. Conclusions

Silver NM300K induced avoidance behaviour in all tested collembolan species. Although not conclusive, it seemed that euedaphic species exhibited more pronounced avoidance behaviour than the epiedaphic species, with the hemiedaphic species ranging somewhere in between. We speculate that this is due to the greater exposure of organisms, e.g., via the furca. From a hazard assessment perspective, it is clear that a single avoidance test with *F. candida* (the standard test species) does not represent the many important collembolan species. Although it was the most sensitive species, we observed a species-specific avoidance behaviour, which ranged over a factor of 50, i.e., from 0.46 to 28 mg Ag/kg with an HC_5_ of approximately 0.39 mg Ag/kg. The present results confirm the importance of preserving biodiversity and that, although large efforts have been made in the past for ecotoxicology with standardized guidelines and representative species, we are currently aware of the limitations. To ensure environmental sustainability, improvements are necessary and possible; indeed, one of the most important is to increase the ecological relevance, e.g., widening the species coverage.

## Figures and Tables

**Table 1:** Morphological and ecological traits of the collembolan species used in the avoidance test.

Species	Family	Length (mm)	Habitat	Furca
*Folsomia candida*	Isotomidae	1.5–2.2	euedaphic	well developed
*Folsomia fimetaria*	Isotomidae	0.8–1.3	euedaphic	well developed
*Proisotoma minuta*	Isotomidae	1–1.2	hemiedaphic	well developed
*Mesaphorura macrochaeta*	Onychiuridae	0.6–0.8	euedaphic	without
*Protaphorura fimata*	Onychiuridae	0.7–1.4	euedaphic	without
*Ceratophysella denticulata*	Hypogastruridae	0.9–2.1	epiedaphic	well developed

**Table 2:** Avoidance effect concentrations (EC_x_, mg Ag NM300K/Kg soil DW) for the different collembolan species when exposed to Ag NM300K in LUFA 2.2 soil (95% confidence intervals).

Species	EC_10_	EC_50_	S	*p*	Habitat Preference
*Folsomia* *candida*	0.46 (0.1–0.9)	12 (8–16)	0.67 (0.74–0.59)	<0.0000	euedaphic
*Folsomia* *fimentaria*	2.3 (0.5–5)	25 (14–36)	1.06 (1.11–1.05)	0.0014	euedaphic
*Proisotoma* *minuta*	3 (3–6)	15 (7–22)	0.75 (0.84–0.63)	0.0012	hemiedaphic
*Mesaprophorura* *macrochaeta*	3 (2–8)	135 (67–202)	0.57 (0.66–0.45)	0.0009	euedaphic
*Protaphorura* *fimata*	8 (5.5–10)	36 (31–41)	1.46 (1.57–1.34)	0.0000	euedaphic
*Ceratophysella* *denticulate*	28 (18–38)	171 (146–196)	1.22 (1.34–1.11)	<0.0000	epiedaphic

## Data Availability

The data presented in this study are available on request from the corresponding author.
